# A Text-Book on Chemistry for the Use of Schools and Colleges

**Published:** 1846-11

**Authors:** 


ART. III.—*A Text-Book on Chemistry for the use of Schools and Colleges.* By JOHN WILLIAM DRAPER, M.D., Professor of Chemistry in the University of New York, Member of the American Philosophical Society, &c. With nearly three hundred illustrations. Harper & Brothers, 82 Cliff street. New York. 1846. pp. 399. 8vo.

This unpretending little volume possesses a much larger share of merit than most of the text-books on chemistry published for the use of schools. The author is a man of genius and learning, and a practical chemist and teacher, who has a thorough knowledge, not only of his subject, but also of the wants of the learner. In the comparatively narrow limits of this work he has succeeded in presenting an intelligible and most interesting outline of the science of chemistry. The selection of facts and illustrations is exceedingly happy, and the work has an originality and freshness about it which would hardly be looked for in such an abridgment. The numerous wood-cuts are executed well, and some of them devised by the author will aid the student materially in comprehending the text. We take great pleasure in recommending Dr. Draper's modest volume to that large class of persons for whom it was prepared, and whose progress in the study of chemistry, we are safe in saying, it is well calculated to accelerate.

